# Supplementary material for: Larger whole brain grey matter associated with long-term Sahaja Yoga Meditation: A detailed area by area comparison
Source: PLoS One. 2020 Dec 28;15(12):e0237552. doi: 10.1371/journal.pone.0237552 (PMC7769288; doi:10.1371/journal.pone.0237552)
Supplement: S1 Appendix — (DOCX) [file pone.0237552.s001.docx]

**S1 Appendix**

As a continuation of what is stated in the Statistical Analysis subsection of the manuscript, this document contains: The classification method in Zones used in order to assign the statistical model at each brain area, with a Figure that helps to understand the decision flow process and two full tables with the statistical data of all areas and their Zone classification: Table S1 with the 16 lobes areas and Table S2 with the 116AAL areas.

**CLASSIFICATION METHOD IN ZONES**

In order to properly understand this section, we recommend the reading of section “Statistical Analysis” in the main manuscript entitled “Increases in whole brain grey matter associated with long-term Sahaja Yoga Meditation: a detailed area by area description”.

**Zones 1, no correlations:** There is no correlation between GMV and TIV nor between GMV and Age, therefore there isn’t any covariate in the model and a t-test of comparison of means is carried out.

$$\mathrm{GMV}_{\mathrm{ij}}= \beta_{0}+\mathrm{Med}_{i}+\varepsilon_{\mathrm{ij}}$$

**Zones 2, correlations with a single covariate:** There is only one correlation between *GMV* and *TIV* or between *GMV* and *Age*. In this case there are four different alternatives for Zones 2.

**Zones 2A and 2B: with significant correlation between *GMV* and *TIV***

- - - **Zones 2A: *Med x TIV* interaction is not statistically significant:** An ANCOVA with *Med* as factor and *TIV* as covariate is carry out.

$${GMV}_{ij}=\beta_{0}+{Med}_{i}+\beta_{2}\cdot{TIV}_{ij}+\varepsilon_{ij}$$

- - - **Zones 2B: *Med x TIV* interaction is statistically significant:** A general linear model analysis with *Med* as factor, *TIV* as covariate and one interaction *Med x TIV* is carry out.

$${GMV}_{ij}=\beta_{0}+{Med}_{i}+\beta_{2}\cdot{TIV}_{ij}+\beta_{3}\cdot\left( Med \times TIV \right)_{ij}+\varepsilon_{ij}$$

**Zones 2C and 2D: with significant correlation between *GMV* and *Age***

- - - **Zones 2C: *Med x Age* interaction is not statistically significant:** An ANCOVA with *Med* as factor and *Age* as covariate is carry out.

$${GMV}_{ij}=\beta_{0}+{Med}_{i}+\beta_{1}\cdot{Age}_{ij}+\varepsilon_{ij}$$

- - - **Zones 2D: *Med x Age* interaction is statistically significant:** A general linear model analysis with *Med* as factor, *Age* as covariate and one interaction *Med x Age* is carry out.

$${GMV}_{ij}=\beta_{0}+{Med}_{i}+\beta_{1}\cdot{Age}_{ij}+\beta_{4}\cdot\left( Med \times Age \right)_{ij}+\varepsilon_{ij}$$

**Zones 3, correlations with a both covariates, *Age* and *TIV*, simultaneously:** In this case there are four different alternatives for Zones 3.

- - - **Zones 3A, none of the interactions *Med x TIV* nor *Med x Age* is statistically significant:** An ANCOVA with *Med* as factor and *TIV* and *Age* as covariates is carry out.
    - ${GMV}_{ij}=\beta_{0}+{Med}_{i}+\beta_{1}\cdot{Age}_{ij}+\beta_{2}\cdot{TIV}_{ij}+\varepsilon_{ij}$
    - **Zones 3B, Only *Med x TIV* interaction is statistically significant:** A general linear model analysis with *Med* as factor, *Age* and *TIV* as covariates and one interaction *Med x TIV* is carry out.
    - ${GMV}_{ij}=\beta_{0}+{Med}_{i}+\beta_{1}\cdot{Age}_{ij}+\beta_{2}\cdot{TIV}_{ij}+\beta_{3}\cdot\left( Med \times TIV \right)_{ij}+\varepsilon_{ij}$
    - **Zonas 3C Only *Med x Age* interaction is statistically significant:** A general linear model analysis with *Med* as factor, *Age* and *TIV* as covariates and one interaction *Med x Age* is carry out.
    - ${GMV}_{ij}=\beta_{0}+{Med}_{i}+\beta_{1}\cdot{Age}_{ij}+\beta_{2}\cdot{TIV}_{ij}+\beta_{4}\cdot\left( Med \times Age \right)_{ij}+\varepsilon_{ij}$
    - **Zones 3D, both interactions *Med x TIV* and *Med x Age* are statistically significant:** A general linear model analysis with *Med* as factor, *Age* and *TIV* as covariates and two interactions *Med x Age* and *Med x TIV* is carry out.

$${GMV}_{ij}=\beta_{0}+{Med}_{i}+\beta_{1}\cdot{Age}_{ij}+\beta_{2}\cdot{TIV}_{ij}+\beta_{3}\cdot\left( Med \times TIV \right)_{ij}+\beta_{4}\cdot\left( Med \times Age \right)_{ij}+\varepsilon_{ij}$$

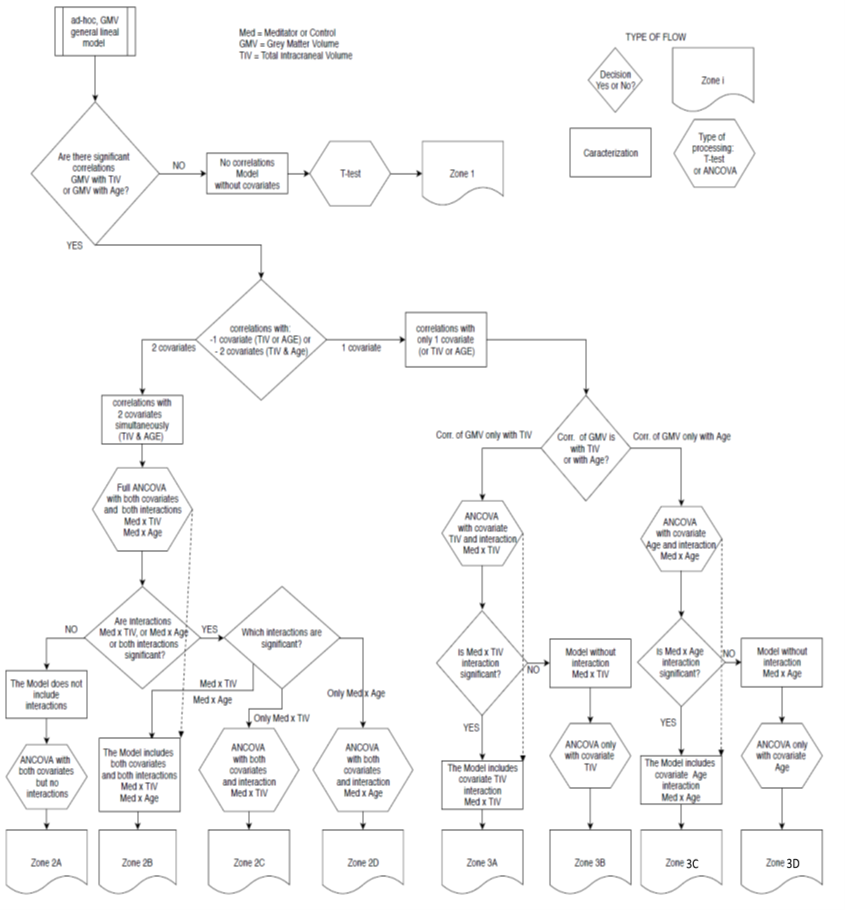


S1 Figure. Flowchart that shows the classification criteria to assign the statistical model to be applied in each brain area, named from Zone1 to zone 3D

From tables S3 Table and S4 Table we can see that the ad-hoc statistical analysis provided that:

- Most areas: 11 out of the 16 lobes subdivision and 71 out of the 116AAL subdivision followed the model named **Zona 3A** in which both Age and TIV covaried significantly with GMV. Also, among the resulting areas this situation was the most predominant: 3 out of the 4 areas at the lobe’s subdivision and all the 11 resulting areas at the AAL’s subdivision.
- Some areas like left and right brainstem, in the 18 lobes areas, or left and right Thalamus among 5 others areas at 116 AAL subdivision followed the simplest model named **Zone 1** where none of the covariates, Age nor TIV was significant.
- The other 29 AAL areas and only one lobe area followed the model **Zone 2A** in which only TIV covaried significantly with GMV, age was not correlated with GMV on these areas.
- 8 AAL areas and none lobe area followed the model **Zone 2B** in which TIV covariates significantly with GMV, and the interaction *Med x TIV was also significant.* Age was not correlated with GMV on these areas.
- And finally, 13 AAL areas and 2 lobes area followed the model **Zone 3B** in which both Age and TIV covaried significantly with GMV and the interaction *Med x TIV was also significant.*
- No area was classified as: Zone 2C, 2D, 3C or 3D
